# Calendar

**DOI:** 10.1155/S1463924687000117

**Published:** 1987


					Journal of Automatic Chemistry ofClinical Laboratory Automation, Vol. 9, No. (January-March 1987), p. 50
Calendar
MARCH
9 to 13 March 38th Pittsburgh Conference and Exposition on
Analytical Chemistry and Applied Spectroscopy: Convention
Center, Atlantic City, New Jersey, USA. Contact Pittsburgh
Conference, Dept. UPD, 12 Federal Drive, Suite 322,
Pittsburgh, Pennsylvania 15235, USA.
12 March Trace Organics in Water: Warrington, UK.
Contact Analytical Division, Royal Society ofChemistry,
Burlington House, London W1V 0BN.
23 to 27 March Short course: Gas Liquid Chromatography:
Loughborough, UK. Contact Dr R. M. Smith, Chemistry
Department, University of Technology, Loughborough,
Leicestershire LE11 3TU, UK.
APRIL
8 to 10 July Short course: Supercritical Fluid Chromatography:
Loughborough, UK. Contact Dr R. M. Smith: (as above).
13 to 18 July 31st International Congress ofPure and Applied
Chemistry: Sofia, Bulgaria. Contact Dr R. Vlahov, Institute
of Organic Chemistry, Bulgarian Academy of Sciences,
1113 Sofia, Bulgaria.
19 to 24 July Annual Meeting ofthe American Association of
Clinical Chemistry: San Francisco, California, USA. Contact
AACC, Office ofContinuing Medical Education, 1725 K
Street N.W., Washington, D.C. 20006.
SEPTEMBER
4 to 6 September IMLS: Quality Assurance Symposium:
Liverpool, UK. Contact Mr D. Kilshaw, Department of
Pathology, Arrowe Park Hospital, Arrowe Park Road,
Upton, Wirral, Merseyside L49 5LN, UK.
14 to 18 September X-ray Spectrometry Conference: Durham,
UK. Contact Maureen Courtney, Pye Unicam Ltd, York
Street, Cambridge CB1 2PX.
3 to 5 April IMLS: Clinical Chemistry/Haematology/Blood
Group Serology Symposium: York, UK. Contact
Mr K. V. Lewis, Pathology Department, Royal Infirm-
ary, Bradford, West Yorkshire BD9 6RJ, UK.
6 to 9 April International Symposium on Electroanalysis in
Biomedical, Environmental and Industrial Sciences: Cardiff
UK. Contact Short Courses Section, UWIST, PO Box 68,
CardiffCF1 3XA, UK.
OCTOBER
4 to 9 October FACCS XIV--1987: Detroit, USA. Contact
Dr S. Jo Swarin, Analytical Chemistry Department,
General Motors Research Laboratories, Warren, Michi-
gan 48090-9055, USA.
12 to 14 October Analyticon 87: London. Contact Scientific
Symposia Ltd, 33/35 Bowling Green Lane, London
EC1R 0DA.
MAY
13 to 15 May Scientific Computing and Automation (Europe):
Amsterdam, The Netherlands. Contact Scientific Computing
and Automation (Europe), c/o Reunion International
BV, Tholenseweg 3, 1181 KD Amstelveen, The Nether-
lands.
JUNE
28June to 4july 13th Annual Congress on Clinical Chemistry:
The Hague, The Netherlands. Contact Dr N. C. den Boer,
PO Box 82000, 2508 EA, The Hague, The Netherlands.
JULY
7 to 10 July Short course: Statistics for Analytical Chemistry:
Loughborough, UK. Contact Dr R. M. Smith (as above).
8 to 10 July Short course: Flow Injection Analysis: Lough-
borough, UK. Contact Dr R. M. Smith (as above).
1988
16 to 21 April 1988 American Society for Clinical Pathology
Spring National Meeting: Kansas City, USA. Contact ASCP,
2100 West Harrison, Chicago, Illinois 60612-37798,
USA.
18 to 21 April Flow Analysis IV: Las Vegas, Nevada, USA.
Contact Professor Gilbert Pacey, Department of Chem-
istry, Miami University, Oxford, Ohio 45056, USA.
24 to 29 July National Meeting ofthe American Association of
Clinical Chemistry: New Orleans, USA. Contact AACC.
National Offices, 1752 K Street N.W., Suite 1010,
Washington, D.C. 20006.
Conference organizers should sendfull details oftheir meetingfor
inclusion in Journal ofAutomatic Chemistry's' calendar at least
three months before the event. Information to the Editor, Journalof
Automatic Chemistry, Taylor & Francis Ltd, 4 John Street,
London WC1N 2ET.
50

				

